# Duodenal Adenocarcinoma in the Setting of Bariatric Surgery: A Perfect Storm for Wernicke’s Encephalopathy

**DOI:** 10.7759/cureus.33765

**Published:** 2023-01-14

**Authors:** Aliza Gross, Allen T Yu, Jacques Lara-Reyna, Koji Park, Eugenius J Harvey

**Affiliations:** 1 Department of Surgery, Icahn School of Medicine at Mount Sinai, New York City, USA; 2 Department of Surgery, Mount Sinai Hospital, New York City, USA; 3 Department of Neurosurgery, University of Illinois College of Medicine Peoria, Peoria, USA

**Keywords:** wernicke encephalopathy, bariatric, gastric band surgery, duodenal adenocarcinoma, thiamine deficiency

## Abstract

Wernicke’s encephalopathy (WE) is a condition resulting from thiamine deficiency that typically presents with acute neurologic symptoms including ataxia, eye movement disorders, and altered mental status. Though classically seen in patients with alcohol use disorder, it can also occur as a complication of bariatric surgery and gastrointestinal cancers. Here, we present a patient with a history of gastric band surgery and an intact alimentary tract. She presented with acute, intractable vomiting and epigastric abdominal pain, incompletely relieved by deflating her gastric band, and was found to have duodenal adenocarcinoma causing partial duodenal obstruction. She was then found to have binocular diplopia, horizontal nystagmus, dizziness, reduced proprioception, and pins-and-needles numbness in her bilateral lower extremities, and there was concern for gait instability; thus, WE was suspected. The patient was treated with high-dose thiamine repletion, and her symptoms resolved shortly thereafter. WE is rare in patients who have undergone gastric band surgery, and to our knowledge, this is the first case of WE in a patient with concurrent duodenal adenocarcinoma. This case illustrates that patients with a history of bariatric surgery may be more susceptible to developing WE in the face of a new gastrointestinal insult, such as duodenal cancer.

## Introduction

Wernicke’s encephalopathy (WE) is an acute neurological sequela of thiamine deficiency, classically associated with the triad of ataxia, disorders of eye movement, and altered mental status. However, the condition presents heterogeneously; less than one-third of WE patients present with this complete triad, and other presenting symptoms may include vertigo, hallucinations, autonomic dysregulation, and seizures [1-5]. Severe thiamine deficiency may also impact the peripheral nervous system and cause pain, numbness, and/or paresthesias, a condition known as dry beriberi that can progress to become WE [6]. While the majority of recognized WE cases present in patients with alcohol use disorder, WE may be caused by any disruption to digestion and nutrient absorption, including gastrointestinal (GI) cancers and bariatric surgery. Unfortunately, WE is only diagnosed in these patients before death an estimated 6% of the time [7]. Currently, Roux-en-Y gastric bypass (RYGB), sleeve gastrectomy (SG), and adjustable gastric banding are the most commonly performed bariatric surgeries [8]. Among the bariatric surgery population, WE most commonly presents in those who have undergone RYGB, but it may also present rarely after surgeries that maintain the luminal surface area of the intestines, such as gastric banding or longitudinal gastrectomy [9]. Gastric adenocarcinoma is the most common cancer that causes WE [10], and there have been no reported cases of WE in a patient with the rare duodenal adenocarcinoma. The unique combination of GI cancer and gastric surgery may leave patients uniquely vulnerable to developing WE. To our knowledge, there have been no reported WE cases in patients with GI cancers who have also undergone gastric band surgery, and there have been no reported WE cases in patients with duodenal adenocarcinoma. Here, we report on a patient who was found to have WE in the setting of duodenal adenocarcinoma and a remote history of gastric band surgery.

## Case presentation

The patient is a female in her early 50s with a past medical history significant only for asthma not requiring treatment. She had a laparoscopic gastric band placement at age 39 when her weight was 158 kg and her height was 1.60 m, with a corresponding BMI of 61.8 kg/m^2^. The patient was lost to follow-up until she presented 10 years after her bariatric surgery to re-establish care. Despite bariatric surgery, she did not have any weight loss; on initial presentation, she weighed 158.3 kg and her nadir weight is unknown. Her daily nutritional intake was on average two meals a day, which consisted of two pieces of buttered toast for breakfast, soup or a sandwich for lunch, and some vegetables for dinner. Serum protein was 7.7 g/dL, and albumin was 4.0 g/dL. She had not adhered to recommended vitamins. She presented complaining of recurrent abdominal pain, shortness of breath, weakness, numbness, and tingling in her hands for the last year. Thus, removal of the gastric band was planned.

**Figure 1:**
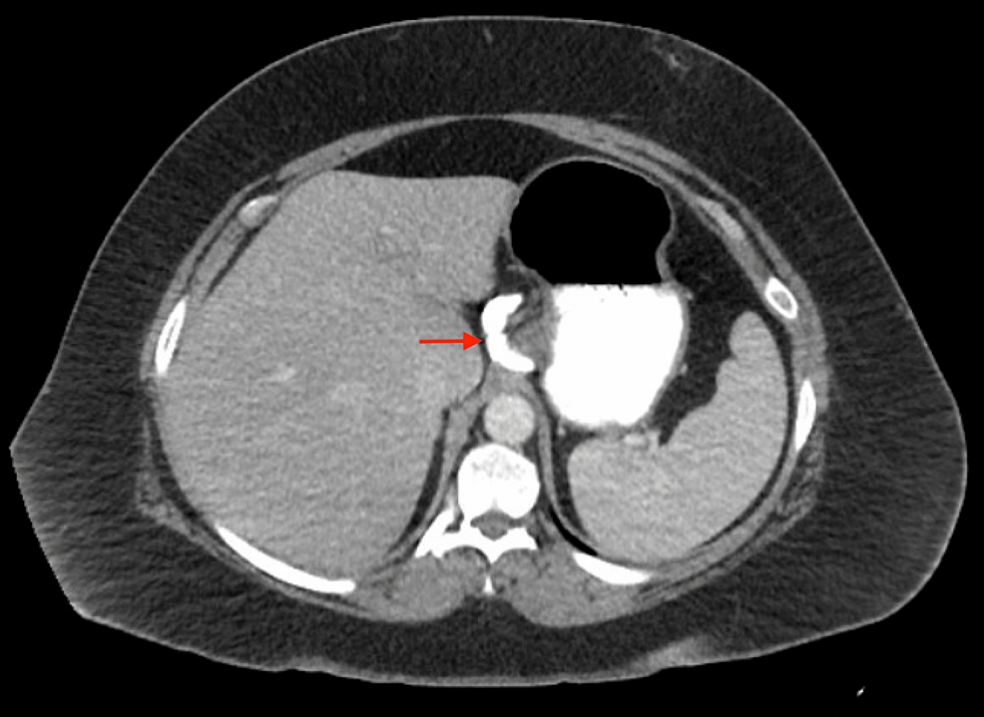
CT abdomen and pelvis with oral and intravenous contrast, with the red arrow pointing to an appropriately placed gastric band.

However, in the interim, she presented to the ED with three weeks of small volume non-bilious, non-bloody vomiting, nausea, dizziness, and a tingling sensation in her feet bilaterally. She had been experiencing epigastric burning abdominal pain that had worsened over the past several weeks. She was not able to go to work due to pain and lethargy. She was unable to tolerate oral foods and could only keep liquids down. Her weight on presentation was not significantly changed from the initial presentation and the initial laboratory workup was within normal limits. Abdominal x-ray and computed tomography (CT) scans demonstrated an appropriately placed gastric band (Figure 1). 

**Figure 2:**
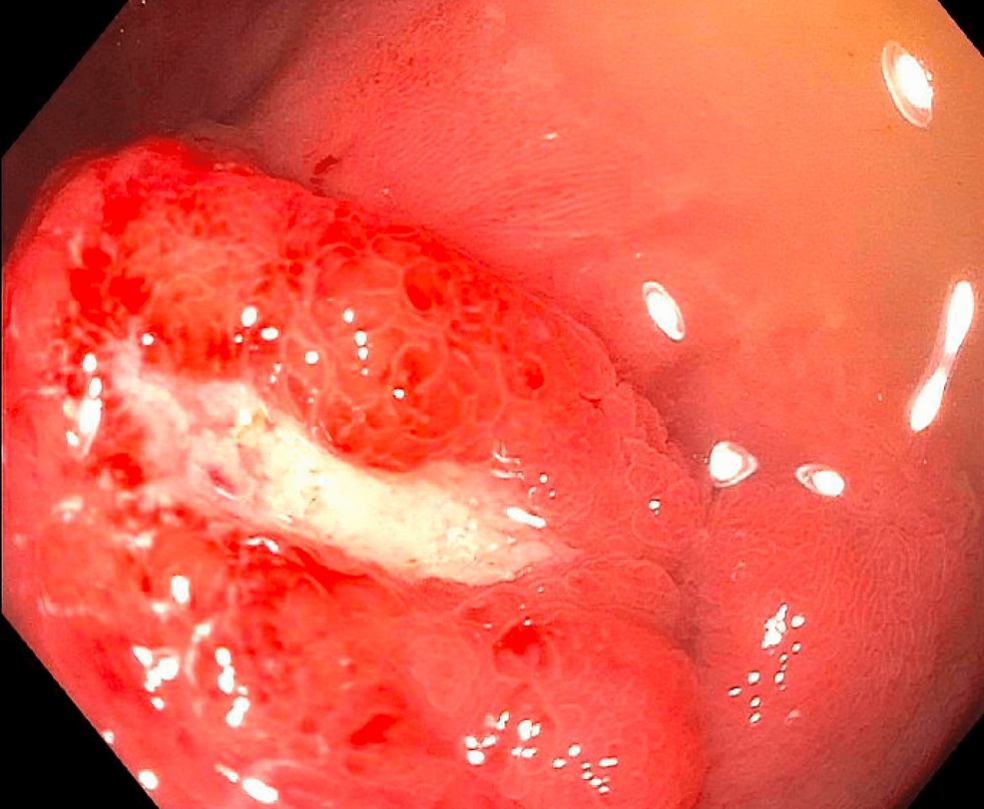
Esophagogastroduodenoscopy showing a duodenal bulb ulcer, later identified on pathology as duodenal adenocarcinoma

She was admitted and had her gastric band completely emptied and deflated. However, while this improved her epigastric pain, she continued to have persistent nausea, vomiting, dizziness, and lower extremity tingling. She was taken for an esophagogastroduodenoscopy and was found to have a minimally bleeding duodenal ulcer likely causing partial gastric outlet obstruction (Figure 2). This ulcer was subsequently identified on pathology as moderately differentiated duodenal adenocarcinoma. She was started on pantoprazole 40 mg IV twice a day. 

**Figure 3:**
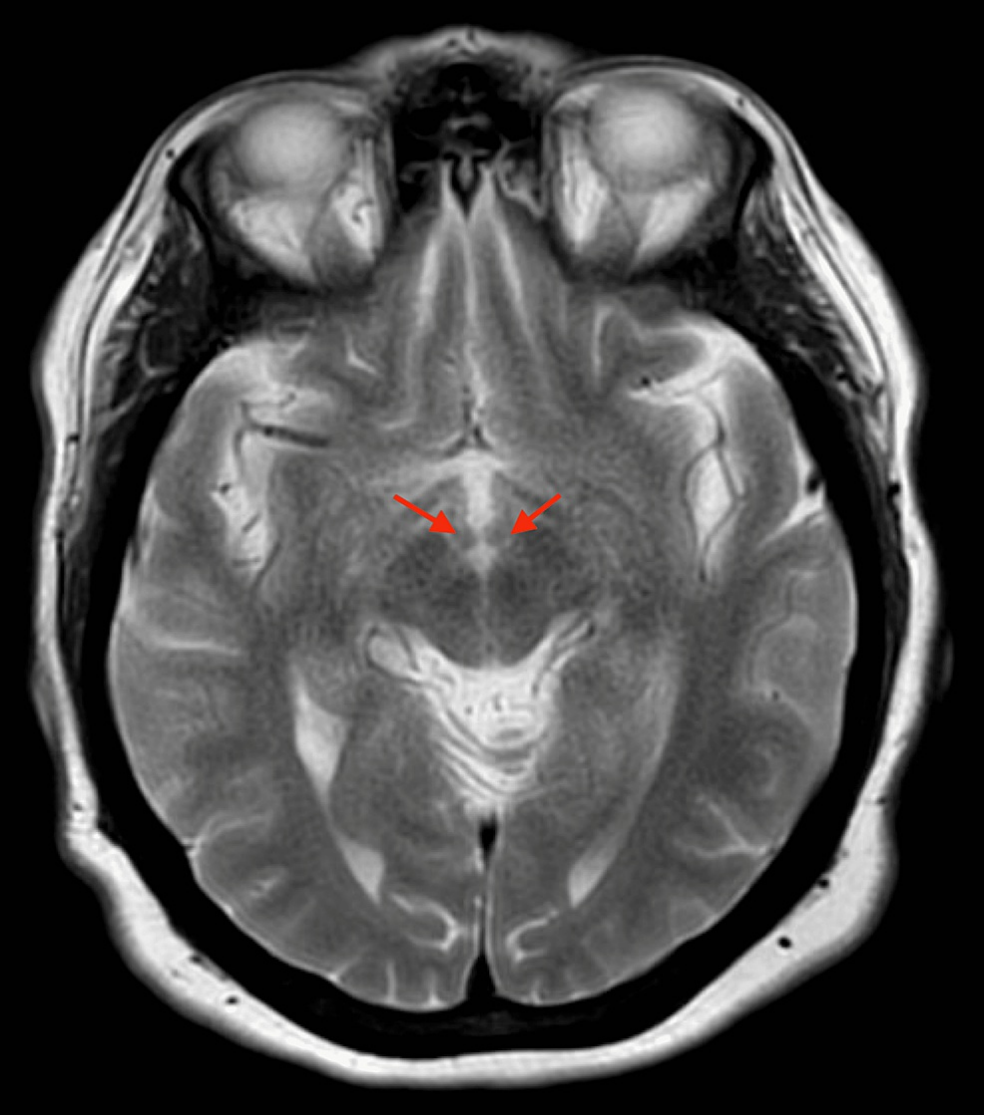
MRI of brain without contrast, T2, axial view. The red arrows are pointing to the bilateral mammillary bodies, which show no hyperintensities. No other abnormal findings were seen on MRI.

Despite adequate hydration with intravenous fluids, on day four of her hospitalization she remained nauseous, began complaining of diplopia, and endorsed worsening of her dizziness. On further questioning, she endorsed intermittent episodes of vertigo at home in the past few weeks and had suffered a fall as well. She denied any tinnitus, hearing loss, seizures, strokes, loss of consciousness, or episodes of diplopia in the past. On her physical exam, her visual acuity was 20/20 bilaterally, pupils were equal, round, reactive to light and accommodation, and visual fields were full to finger counting. Facial strength, sensation, and hearing were all within normal limits. Her exam was significant for a horizontal nystagmus with a left rotary component when looking down. She had slightly reduced proprioception in her toes compared to her fingertips, and pins-and-needles numbness was endorsed in the bilateral lower extremities. The patient’s gait and station examination was deferred due to her dizziness and vertigo; thus, it was unclear if vertigo or ataxia is what caused her fall. Concern for early-stage WE was heightened, and the neurology service was consulted. MRI of the brain was performed, and no hyperintensities of the mammillary bodies were seen (Figure 3). She was started on high-dose thiamine repletion with 500 mg intravenously three times daily for two days, 250 mg intramuscularly daily for five days, and then 100 mg orally daily indefinitely. The day after initiating treatment, her dizziness, diplopia, and nausea largely resolved. Additional workup for micronutrient deficiencies and endocrine imbalances revealed low vitamin B12 levels (< 148 pg/mL), low vitamin D levels (5.2 ng/mL), presence of anti-thyroid peroxidase (TPO) antibodies, elevated thyroid-stimulating hormone (TSH) (21.872 uIU/mL), and low free T4 (0.69 ng/dL). She was discharged on hospital day seven with high-dose vitamin B12, thiamine, and vitamin D, as well as with levothyroxine 100 mcg daily. Her duodenal adenocarcinoma was later found to be stage IV on diagnostic laparoscopy. She has since been receiving palliative chemotherapy and total parenteral nutrition, with oral feeds as tolerated. 

## Discussion

WE, while typically described in patients with alcohol use disorder, is becoming increasingly recognized in the setting of a wide variety of GI tract pathologies, including bariatric surgeries and GI cancers. WE after bariatric surgery is a rare but serious complication of bariatric surgery; at least 118 cases have been reported to date [9]. WE is a clinical diagnosis, but its presenting symptoms can vary widely. Patients with WE in the setting of bariatric surgery most commonly present with ataxia (84.7%), while cancer patients who develop WE are more likely to present with altered mental status (90%) [11]. However, it is important to note that about 19% of patients with WE may not present with any of the classic triad symptoms [12]. Of note, our patient's chief neurological complaint was dizziness and vertigo. While not classically identified as a WE symptom, many cases of WE also have exhibited vertigo and vestibulopathy, especially in those with non-alcoholic WE [2-5]. Untreated, WE may cause the potentially irreversible Korsakoff psychosis, and may even cause death in 20% of patients [1]. Patients with nonalcoholic WE are often diagnosed much later with more severe symptoms than patients with alcohol use disorder, despite patients with nonalcoholic WE being more likely to seek medical care for their symptoms [11]. Thus, it is of the utmost importance for physicians to maintain high clinical suspicion of WE in any patient with neurological symptoms in the setting of any risk factors for malnutrition, including bariatric surgery, prolonged intractable vomiting, and GI cancers.

Bariatric surgery patients are at particular risk for micronutrient deficiencies. In fact, it is estimated that 18% of bariatric surgery patients have thiamine deficiency [13]. RYGB accounts for 52% of post-bariatric surgery WE cases, followed by sleeve gastrectomy at 21% [9]. However, WE may also occur after surgeries where GI anatomy and absorptive capacity are left intact, such as gastric band surgery. There have been 6 reported cases of WE after gastric band surgery, not including our patient, in the past 37 years [9]. WE, in these patients, is usually brought on by poor oral intake or prolonged vomiting. While most cases of WE occur in the months following bariatric surgery, several cases have been reported years later, including two cases reported six and 13 years, respectively, after gastric banding [9]. Severe vomiting has been found to precede WE in bariatric surgery patients in more than 87% of cases [13]. 

GI cancers have also been recognized independently as a risk factor for developing WE. GI tract cancers may lead to WE by causing decreased oral intake, chronic vomiting, and GI tract damage leading to malabsorption. Cancers may also rapidly consume thiamine, reducing the body’s existing stores and causing further thiamine depletion [14]. However, WE is most commonly seen in gastric cancer, followed by advanced colorectal cancer [10,15]. To our knowledge, our patient is the first to develop WE with duodenal adenocarcinoma. Malignancies of the small bowel are already highly uncommon, accounting for only 3% of all GI cancers [16]. WE is both a rare occurrence and a diagnosis often missed, especially in patients with non-alcoholic risk factors. In a multinational meta-analysis of autopsy studies, 94% of patients without alcohol use disorder who were found to have WE postmortem were not diagnosed antemortem [7]. These reasons together could explain why a case of WE in a duodenal adenocarcinoma patient has not been reported in the literature thus far.

In our patient, acute severe vomiting due to duodenal adenocarcinoma in the setting of chronic malnutrition from gastric band surgery ultimately led to the development of WE. Though our patient’s gastric band surgery was completed 14 years ago, there has been one reported case of WE in a patient with a history of gastric band surgery 13 years prior, suggesting that even years after surgery, gastric band surgery patients may carry increased susceptibility to developing thiamine deficiencies [9]. Thirty percent of bariatric surgery patients are suspected of having subclinical thiamine deficiency and, under new stressors, these patients are prone to develop symptoms of acute thiamine deficiency [17]. Our patient was lost to follow-up and was not adherent to post-operative nutritional supplementation, making it highly likely that she had underlying subclinical thiamine deficiency. Thus, she was highly vulnerable to developing WE in the face of any new precipitating factor and, in her case, it was duodenal adenocarcinoma causing partial obstruction and intractable vomiting. 

All bariatric surgery patients should receive daily thiamine supplementation post-surgery for thiamine-deficiency prophylaxis. The American Society for Metabolic and Bariatric Surgery recommends that post-bariatric surgery patients with certain high-risk characteristics, including new GI symptoms such as intractable nausea and vomiting, receive thiamine screening at least every three to six months [18]. If thiamine deficiency is expected, clinicians should treat the deficiency empirically and without laboratory confirmation, as the consequences of not treating thiamine deficiency early are grave and thiamine is safe and non-toxic. Treatment of suspected WE or acute thiamine deficiency should consist of high-dose parenteral thiamine supplementation until symptom resolution, followed by 100 mg of oral thiamine daily indefinitely [18]. 

## Conclusions

This report presented a rare case of WE in a patient with duodenal adenocarcinoma with likely underlying subacute thiamine deficiency from her history of gastric band surgery with non-adherence to post-operative nutritional supplementation. WE may be underrecognized or diagnosed later in the disease course in patients with these risk factors, compared to patients who develop WE in the setting of alcohol use disorder. It may present heterogeneously and may not always present with the classic triad of ataxia, nystagmus, and dementia. Therefore, clinical suspicion should remain high for WE in patients presenting with neurologic symptoms, especially in patients with a history of gastric band surgery, GI cancers, or intractable vomiting. 

## References

[1] Ota Y, Capizzano AA, Moritani T, Naganawa S, Kurokawa R, Srinivasan A (2020). Comprehensive review of Wernicke encephalopathy: pathophysiology, clinical symptoms and imaging findings. Jpn J Radiol.

[2] Kattah JC (2020). Early signs of thiamine deficiency: a case report. Ann Intern Med.

[3] Oudman E, Wijnia JW, Oey MJ, van Dam MJ, Postma A (2018). Preventing Wernicke's encephalopathy in anorexia nervosa: a systematic review. Psychiatry Clin Neurosci.

[4] Okafor C, Nimmagadda M, Soin S, Lanka L (2018). Non-alcoholic Wernicke encephalopathy: great masquerader. BMJ Case Rep.

[5] Wiggli B, Kapitza S, Ahlhelm F, Tarnutzer AA (2020). Early recognition of thiamine deficiency: ocular motor deficits in a patient with nutritional deprivation due to persistent antibiotic-related nausea. Infection.

[6] Shible AA, Ramadurai D, Gergen D, Reynolds PM (2019). Dry beriberi due to thiamine deficiency associated with peripheral neuropathy and Wernicke's encephalopathy mimicking Guillain-Barré syndrome: a case report and review of the literature. Am J Case Rep.

[7] Galvin R, Bråthen G, Ivashynka A, Hillbom M, Tanasescu R, Leone MA (2010). EFNS guidelines for diagnosis, therapy and prevention of Wernicke encephalopathy. Eur J Neurol.

[8] Mulita F, Lampropoulos C, Kehagias D (2021). Long-term nutritional deficiencies following sleeve gastrectomy: a 6-year single-centre retrospective study. Prz Menopauzalny.

[9] Oudman E, Wijnia JW, van Dam M, Biter LU, Postma A (2018). Preventing Wernicke encephalopathy after bariatric surgery. Obes Surg.

[10] Kudru CU, Nagiri SK, Rao S (2014). Wernicke's encephalopathy in a patient with gastric carcinoma: a diagnosis not to miss. BMJ Case Rep.

[11] Oudman E, Wijnia JW, Oey MJ, van Dam M, Postma A (2021). Wernicke-Korsakoff syndrome despite no alcohol abuse: a summary of systematic reports. J Neurol Sci.

[12] Sechi G, Serra A (2007). Wernicke's encephalopathy: new clinical settings and recent advances in diagnosis and management. Lancet Neurol.

[13] Lange J, Königsrainer A (2019). Malnutrition as a complication of bariatric surgery - a clear and present danger?. Visc Med.

[14] Kim KH (2013). Wernicke-Korsakoff syndrome in primary peritoneal cancer. Case Rep Oncol.

[15] Jung ES, Kwon O, Lee SH (2010). Wernicke's encephalopathy in advanced gastric cancer. Cancer Res Treat.

[16] Siegel RL, Miller KD, Fuchs HE, Jemal A (2022). Cancer statistics, 2022. CA Cancer J Clin.

[17] Wilson RB (2020). Pathophysiology, prevention, and treatment of beriberi after gastric surgery. Nutr Rev.

[18] Parrott J, Frank L, Rabena R, Craggs-Dino L, Isom KA, Greiman L (2017). American Society for Metabolic and Bariatric Surgery integrated health nutritional guidelines for the surgical weight loss patient 2016 update: micronutrients. Surg Obes Relat Dis.

